# Strategies for Improving Small-Molecule Biosensors in Bacteria

**DOI:** 10.3390/bios12020064

**Published:** 2022-01-25

**Authors:** Corwin A. Miller, Joanne M. L. Ho, Matthew R. Bennett

**Affiliations:** 1 Department of Biosciences, Rice University MS-140, 6100 Main St., Houston, TX 77005, USA; Corwin.A.Miller@rice.edu (C.A.M.); joanne.ho@rice.edu (J.M.L.H.); 2 Department of Bioengineering, Rice University MS-140, 6100 Main St., Houston, TX 77005, USA

**Keywords:** whole-cell biosensor, bacterial biosensor, biosensor engineering, protein engineering, genetic engineering, genetic circuits

## Abstract

In recent years, small-molecule biosensors have become increasingly important in synthetic biology and biochemistry, with numerous new applications continuing to be developed throughout the field. For many biosensors, however, their utility is hindered by poor functionality. Here, we review the known types of mechanisms of biosensors within bacterial cells, and the types of approaches for optimizing different biosensor functional parameters. Discussed approaches for improving biosensor functionality include methods of directly engineering biosensor genes, considerations for choosing genetic reporters, approaches for tuning gene expression, and strategies for incorporating additional genetic modules.

## 1. Introduction

The characterization of the bacterial Lac repressor *lacI* proved a watershed moment in molecular biology. First detailed in 1961, these studies described how the Lac repressor responds to lactose and isopropyl β-*d*-1-thiogalactoside (IPTG) to regulate the expression of genes within the *lac* operon [1]. While this work is best known as an examination of the mechanisms of genetic regulation [2,3], it also showed for the first time how a cell-based system can be controlled through the addition of a small-molecule inducer. Since then, a wide variety of additional bacterial biosensors have been identified, capable of producing a genetic output in response to small molecule analytes [4,5]. Such microbial biosensors have been shown to be capable of serving as a highly effective detection method and producing highly sensitive and specific analyte detection while remaining cheap and easy to produce.

In recent years, biosensors have been used for an increasing number of applications. Generally, we can classify these applications as relating to either industrial, medical, or environmental detection. Within industry, biosensors provide a potent tool for protein and metabolic engineering, allowing a pathway or nutrient to be monitored in real time [6,7] and also enabling screening of large numbers of mutant variants [8]. Biosensors also provide a useful tool for drug screening, providing both a rapid and highly specific response to a library of candidate molecules [9,10]. In a medical context, bacterial biosensors can be used either in situ or within a test tube to detect a variety of ailments, including cancer [11,12], gut inflammation [13], and infection [14,15,16]. For example, the output of a biosensor within bacteria can be tailored to not only detect but also respond to different disease states, to generate “living bacterial therapeutics” [11,12,16,17]. For environmental detection, biosensors have been used to identify contaminants within soil samples, water samples, waste streams, and a variety of other contexts [4,17,18,19]. Contaminant analytes that can be detected by biosensors include heavy metals [20,21], organic contaminants [17,22,23], and pharmaceuticals [9,24,25,26]. Whole-cell biosensors are often advantageous in environmental contexts, as they often require relatively little instrumentation and thus enable “on-site” detection.

**Figure 1:**
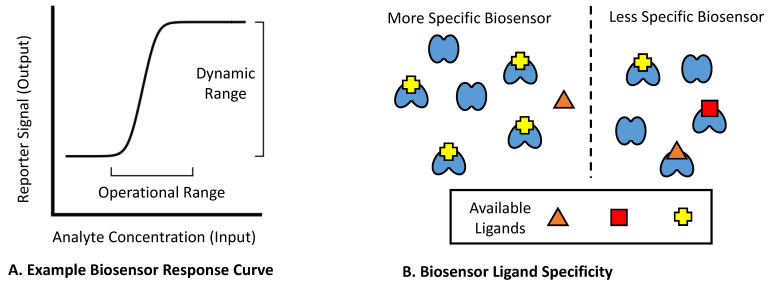
Functional characteristics of genetic biosensors. (**A**) A biosensor’s operational range describes the input range of analyte concentrations over which the sensor produces a detectable change in output. The dynamic range of a biosensor in turn refers to the range of output signal over which the biosensor produces a detectable change in response to analyte ligands. Typically, a biosensor’s dynamic range is described by its fold-induction (also known as the signal-to-noise ratio), which is calculated by dividing the biosensor’s highest measured output by its lowest measured output. (**B**) A biosensor’s specificity refers to the range of distinct analyte compounds to which it is capable of producing a response, with more specific biosensors responding to fewer ligands.

For many existing biosensors, their use in practical applications can often be limited by poor functionality. Generally, three attributes can be used to describe a genetic biosensor’s function: (1) the operational range relative to its input, (2) the dynamic range of its output, and (3) the chemical specificity of the analytes to which it responds (Figure 1). A biosensor’s operational range is defined by the analyte concentrations that produce a response, with relevant features including the lowest analyte concentration that yields a detectable response, and the maximal analyte concentration that yields a saturated response [27]. The dynamic range of a biosensor (also referred to as its fold-induction or signal-to-noise ratio) evaluates the quantity of the output signal at the highest versus the lowest detectable analyte concentration [28]. Lastly, a biosensor’s specificity pertains to the range of analytes it can respond to, with less specific biosensors responding to a wider range of chemicals [29,30]. To date, numerous techniques have been devised to improve each of the above attributes. As the type of desired parameters depends upon the specific application in question, it is frequently necessary to retune or engineer a biosensor to tailor it to a given project. As these biosensor attributes are interdependent, they typically cannot be tuned independently [31], with this feature often complicating engineering efforts.

While whole-cell microbial biosensors are the focus of this review, we note here that numerous other biosensors have been described that incorporate purified proteins or nucleic acids in vitro [5,6]. These types of sensors often utilize an immobilized purified ligand-binding biological element, such as an antibody, aptamer, receptor, or lectin [32]. Purified enzymes can also be used in a similar fashion [33], as demonstrated by the landmark description of a glucose biosensor by Clark and Lyons [6]. The interaction between this immobilized element and an analyte ligand is then detected using a transducer element, which produces a readout signal. Many frequently used transducer modules detect ligand-induced changes within the electrical environment, for instance, monitoring electrochemical, amperometric, voltametric, conductometric, impedimetric, or potentiometric properties [34,35,36,37]. Transducers that monitor optical changes are also common, including interferometry, photonic resonance, and plasmonic resonance-type devices [34,38,39,40]. In additional to electrical and optical transducers, prior studies have also utilized acoustic, mechanical, thermal, and magnetic types of transducers [34,35]. The use of such sophisticated analytical techniques enables detection without a dedicated biological reporter element, and is in part facilitated by the purification of biological components from the complex cellular milieu. Compared to cell-based biosensors, the use of purified components can thus prove particularly advantageous for instances where a biological recognition event cannot readily be linked to a reporter gene. In contrast, detection of a cellular reporter gene typically requires simpler and more commonplace laboratory equipment (see Section 3.3), and thus may be preferable for projects in which engineering a genetic linkage is more tractable. Notably, both types of biosensors are capable of highly specific and sensitive detection, with sensitivity typically on the nanomolar scale (depending on the affinity of the recognition element) [5].

## 2. Mechanistic Classes of Biosensors within Bacterial Cells

### 2.1. Known Mechanisms of Bacterial Biosensors

**Figure 2:**
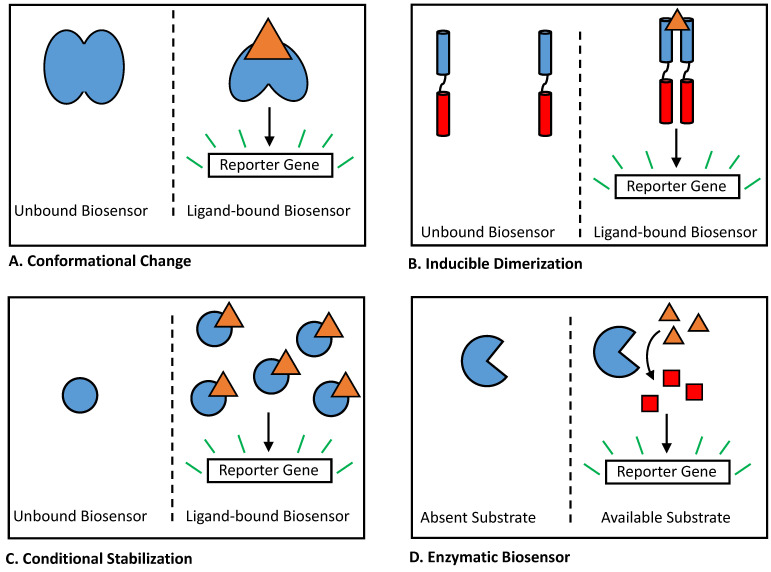
Known mechanisms of bacterial biosensors. (**A**) Some biosensors undergo an allosteric conformational change after binding to a ligand. The biosensor’s adoption of the ligand-bound conformation thereby results in activation of regulated genes. (**B**) For other biosensors, recognition of an analyte by a ligand-binding domain can cause a normally monomeric sensor to dimerize. Dimerization in turn results in activation of an effector domain, leading to activation of regulated genes. (**C**) A third class of bacterial biosensor relies on an activator protein that is unstable and rapidly degraded in the absence of ligand. After ligand binding, the stability of the biosensor is improved, resulting in an increased steady-state protein concentration and greater activation of regulated genes. (**D**) Enzymes can also be used as biosensors in bacteria. Enzymatic biosensors chemically convert their otherwise undetectable substrate into a newly detectable compound.

When developing a strategy to utilize or engineer a biosensor, it is often necessary to consider its detection mechanism. Generally, genetically encoded biosensors function by regulating the expression or activity of a reporter gene, which in turn produces a detectable output signal. Although a wide variety of whole-cell biosensors have been studied in bacteria, they can mostly be grouped within a small number of mechanistic categories based upon how they respond to an analyte. To date, four types of biosensor response mechanisms have been previously described: conformational change, induced dimerization, conditional stabilization, and conditional enzymatic reaction (Figure 2).

### 2.2. Biosensors Reliant on Conformational Change

Among bacterial biosensors, perhaps the most studied mechanism for regulating a reporter gene is for the biosensor to undergo an allosteric or conformational change in the presence of its ligand (Figure 2A). This mechanism is widely used by one-component bacterial transcription factors, including sensors within the LacI family [41], TetR family [42], GntR family [43], and IclR family [44]. In nature, these families of transcription factors all control the expression of regulated genes through a mechanism of de-repression. In the absence of ligand, the transcription factor binds a DNA operator site, blocking transcription of downstream genes. When an inducer ligand is present, the transcription factor binds the ligand and undergoes a conformational shift, leading to the release of DNA and the activation of transcription. While the majority of biosensors using this mechanism have been identified from nature, to date, several small-molecule biosensors relying on a conformational shift have been developed in the lab. For many of these sensors, Förster resonance energy transfer (FRET) is used as a detectable output [8,45]. These sensors often use two fluorescent proteins, such as cyan fluorescent protein (CFP) and yellow fluorescent protein (YFP), together as an FRET pair, relying on the conformational change of the biosensor protein to bring both proteins into closer proximity in the presence of ligand [46,47,48,49]. In a recent work by Juárez et al., a synthetic transcription factor biosensor was constructed in the lab, linking ligand binding to DNA transcription through an engineered allosteric interaction [50]. Developing a new biosensor with a mechanism of this complexity represents a significant challenge, and recent success in this area may represent a promising direction for future work within the field.

In contrast to transcription factor proteins, riboswitches are instead composed of RNA; however, they function as biosensors through a similar mechanism [51,52]. Upon the addition of a ligand, the aptamer domain of a riboswitch binds to it. This binding event in turn leads to a conformational change within the effector domain of the riboswitch, resulting in the activation of downstream genetic elements. In nature, riboswitches typically regulate promoters (controlling transcription) or ribosome-binding sites (controlling translation initiation) [53,54], effectively blocking gene expression in the absence of ligand, while allowing gene expression upon ligand binding. In recent years, artificial RNA sensors (termed ‘aptazymes’) have been developed in the laboratory that instead link ligand binding to the activity of a ribozyme [55,56]. Aptazymes can provide several advantages compared to traditional riboswitches and represent an exciting avenue of biosensor research.

### 2.3. Biosensors Utilizing Inducible Dimerization

Inducible dimerization represents a second common biosensor mechanism in bacteria (Figure 2B). For biosensors of this type, a ligand-binding domain binds an analyte as a dimer, whereas in the absence of ligand, it is more likely to be present in a monomeric form. This binding event also indirectly promotes the dimerization of a second effector domain, which requires dimerization to mediate the activation of regulated genes. In bacteria, two-component signaling systems provide the best-known example of this mechanism. Two-component systems typically contain a transmembrane sensor protein and its associated cytosolic response regulator protein [57,58]. When a ligand comes into contact with the cell, it is recognized by the extracellular-facing ligand-binding domain of the sensor protein. This event promotes dimerization of the sensor protein, resulting in activation of histidine kinase activity and loss of phosphatase activity within its cytosolic-facing domain [57]. This activation in turn leads to increased phosphorylation of the associated response regulator protein, which then changes the conformation and stimulates the expression of regulated genes [59]. Single-component bacterial biosensors using a similar mechanism have recently been constructed in the laboratory, using inducible dimerization of a transmembrane protein to build sensors that increase gene expression in response to caffeine [60] and bile salts [61]. Nucleic acids can also be used to build biosensors that rely on ligand-dependent dimerization, with various split aptamers [62,63], ribozymes [64,65], DNAzymes [66], and aptazymes [67,68] having been built to date. However, despite the relative abundance of existing aptamers, reliably splitting an aptamer or a catalytic nucleic acid element without abolishing its function remains a significant challenge [69].

### 2.4. Conditionally Stabilized Biosensors

Conditional stabilization provides a third biosensor mechanism commonly seen in bacteria (Figure 2C). This type of biosensor typically functions as a transcriptional activator, which, in the absence of ligand, is degraded quickly within the cell. Upon binding to its ligand, the biosensor becomes stabilized, thereby increasing its steady-state concentration and resulting in greater activation of transcription. The best-known natural examples of this mechanism in bacteria lie within the LuxR family of quorum sensors [70]. Multiple biosensors within this family have been shown to depend upon ligand binding for correct protein folding, exhibiting poor solubility and rapid degradation in its absence compared to improved stability and activity upon the addition of ligand [71,72,73]. In recent years, researchers perhaps unintentionally mimicked the natural sensing strategy of LuxR proteins in separate studies developing synthetic biosensors that utilize an analogous mechanism to respond to macrolide antibiotics [74], progesterone [75], auxin [76], and fentanyl [26]. These sensors each contain a ligand-binding domain that has been engineered to be destabilized in the absence of ligand. These domains are then fused to transcriptional activator domains, to enable reduced degradation of the fusion protein and increased activation activity in the presence of ligand. While the first of these four studies was performed in *E. coli*, the latter three studies were performed in yeast [75] and Arabidopsis [26,76], respectively, demonstrating the versatility of this design strategy across different domains of life.

### 2.5. Enzymatic Biosensors

Lastly, chemical or enzymatic reaction of a biosensor with its ligand can be viewed as a fourth type of bacterial sensor mechanism (Figure 2D), though such sensors are considerably less common and their functionality less uniform than the three aforementioned types. Use of this strategy was described in 2015 by Libis et al. [77] to detect several compounds of interest. In this work, the authors added exogenous metabolic enzymes to *Escherichia coli* cells that mediated the conversion of target analytes into detectable ligands for existing biosensors, producing strains capable of sensing cocaine, nitroglycerin, chlorpropham, 2-chloro-4-nitrophenol, parathion, and hippurate [77]. Other groups have since utilized metabolic enzymes in a similar fashion, producing biosensor strains to detect a variety of compounds, including lignin [78], glycerate [79], phenylalanine [80], and methanol [81]. In addition to metabolic enzymes, biosensors have also been constructed using aminoacyl tRNA synthetase (aaRS) proteins, a class of enzyme that catalyzes ligation between an amino acid ligand and its cognate tRNA, which leads to incorporation of the amino acid into proteins. To date, aaRS enzymes have been used in *E. coli* cells to detect isoleucine [82], pyrrolysine [83], and a wide variety of synthetic amino acid derivatives [84,85,86,87]. In prior studies, both metabolic and aaRS enzymes have been used to link analyte ligands either to reporter genes or directly to cell growth. In the latter approach, biosensing *E. coli* strains are made to be auxotrophic for the analytes of interest, with this strategy having been previously employed to detect both naturally occurring and xenobiotic compounds [88,89,90].

## 3. Methods for Improving Bacterial Biosensor Properties

### 3.1. Direct Engineering of Biosensor Genes

**Figure 3:**
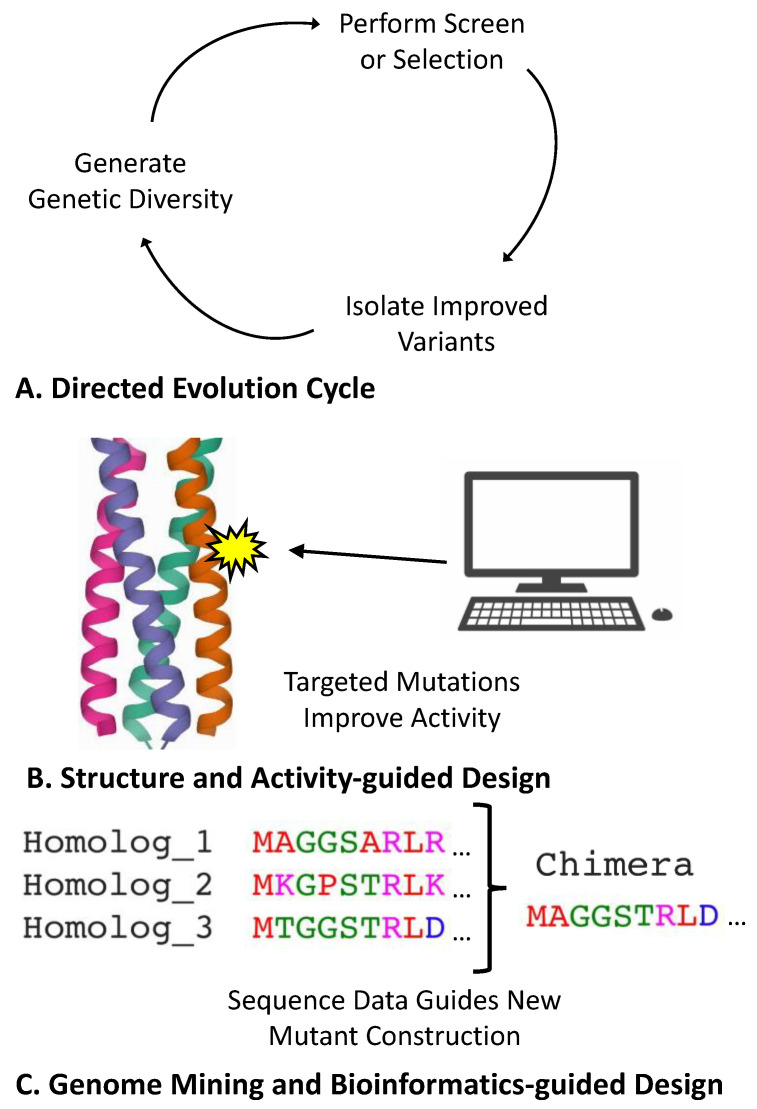
Common methods for direct engineering of biosensor genes. (**A**) Biosensor genes are often improved using directed evolution. In this cyclical approach, a diverse mutant library is first generated from an initial biosensor gene. A selection or screen is then used to measure the activity of variants within the library, and mutants exhibiting improved properties are then isolated. These mutants can then be used to seed subsequent rounds of evolution. (**B**) Prior data regarding a biosensor’s structure or activity can also be used to guide engineering efforts. This approach relies on introducing targeted mutations using an informed prediction of a protein’s properties. Computational approaches and simulations can help guide these efforts. (**C**) Rational design efforts can also utilize DNA sequence data to guide biosensor engineering. In this strategy, homologous or related protein variants are typically identified within genome sequence databases using bioinformatic alignments. This data is then used to guide the construction of mutant or chimeric variants of the gene of interest.

A common route for improving a biosensor’s properties is to directly engineer the protein or RNA sensor component. While this approach often requires significant attention be devoted to individual biosensors, the plethora of available protein engineering techniques makes it possible to improve any of the core properties detailed in Figure 1. Among the protein engineering techniques, directed evolution provides a particularly versatile approach for biosensors (Figure 3A). Directed evolution first entails making a diversified library of a gene of interest, using either targeted or random mutagenic methods. This library is then subjected to a screen or selection to isolate mutant variants with improved properties [91]. While it can often be a challenge to link a gene of interest to a screen or selection marker [92], biosensors often simplify this task as their intrinsic mechanism entails a detectable output. Directed evolution has previously been used to alter the specificity profiles of several biosensors, including the arabinose biosensor AraC [93], the lactose biosensor LacI [94], the erythromycin biosensor MphR [9], and the caffeine riboswitch biosensor CaffRS [95]. Similarly, directed evolution has also been used to improve the dynamic and operational range of biosensors, including the muconic acid biosensor BenM [96], the aromatic aldehyde biosensor PcaV [97,98], and the vanillic acid biosensor VanR [98]. This approach has also been used to improve RNA-based riboswitches [99], including sensors for theophylline [100] and thiamine pyrophosphate [101]. In contrast to rational engineering methods (discussed below), directed evolution can be performed without the need for significant prior knowledge regarding a biosensor of interest. However, depending on the improvements desired and the tractability of a given biosensor system, successful directed evolution projects can often require more significant effort within the lab. Additionally, while directed evolution often readily allows improvement of a biosensor’s dynamic and operational range, evolved extensions to a sensor’s specificity are often limited to compounds that are chemically related to its original ligand.

Structure- and activity-guided protein design provides another route for engineering biosensors (Figure 3B). These methods fall under the umbrella of rational design, wherein detailed knowledge of a protein of interest is leveraged to make targeted mutations to change a protein’s activity [102,103,104]. For structure-guided design, researchers must begin by first obtaining a detailed protein structure. Traditionally, structures are determined experimentally using X-ray crystallography or alternative methods, such as cryo-electron microscopy (cryo-EM) and two-dimensional nuclear magnetic resonance (2D-NMR); however, the recent development of the AlphaFold algorithm raises the possibility of obtaining highly accurate protein structures through computational prediction alone [105]. Once elucidated, structures are used to predict how different mutations can alter a protein’s activity, often aided by sophisticated computational models or simulations [106,107]. Laboratory studies on a given protein’s activity provide a second source of useful information, with researchers often making targeted mutations to identify important residues or test predictions made using structural models. This strategy has been used in the development of several small-molecule biosensors, including sensors for fentanyl [26], TNT [108], and digoxigenin [109]. Structure and activity information can also be used to identify targeted residues for directed evolution (termed semi-rational design), with prior applications of this approach including improvements to the cationic amino acid biosensor LysG [110], the choline biosensor BetI [111], and the tetracycline biosensor TetR [112]. Compared to directed evolution, rationally guided methods can often be more adept at generating new biosensor functionality by linking ligand-binding and output effector domains. Conversely, directed evolution approaches can frequently provide a more effective or expedient route for optimizing an existing functional attribute, though rationally guided methods may be preferable for optimizing systems that have previously undergone extensive characterization.

Genomic mining and bioinformatically guided methods also serve as an effective strategy for biosensor engineering (Figure 3C). Though genomic mining is also considered a rational engineering strategy, this approach relies on DNA sequence information instead of knowledge regarding protein structure–function properties. In genomic mining, DNA sequence databases are first queried to bioinformatically identify natural variants or homologs of a protein of interest [113,114]. These variants can then be directly tested in the laboratory or used to introduce mutations to or create chimeras with the original protein of interest. The efficacy of this approach has benefited greatly from the advent of next-generation sequencing technologies, as the increasing number of available microbial genome sequences enables the rapid identification of numerous testable homologs [113,114]. Genomic mining has proven remarkably effective at identifying new biosensors in nature, enabling the development of sensors for stilbenes [115], β-alanine [116], and progesterone [117]. Bioinformatic approaches have also been used to construct chimeras to combine properties amongst biosensors within related families, with this approach having been applied to both two-component systems [118,119] and allosteric transcription factors [120]. Compared with directed evolution methods, genomic mining typically provides a more facile approach for identifying biosensors with new functionality, and it can often provide a faster route for significantly altering a biosensor’s specificity profile. Bioinformatic strategies also provide a useful method of rational design for riboswitch engineering, as sequence information for many such RNA elements is often available in instances where structural data is unavailable [99]. Similar to other rational design approaches, however, improvements garnered from genomic mining can be limited by the available data. Notably, while the exploration of gene variants found within nature can often turn up surprising results, genomic mining approaches can prove less suitable for projects concerned with synthetic or non-natural analytes.

### 3.2. Optimization of Gene Expression

**Figure 4:**
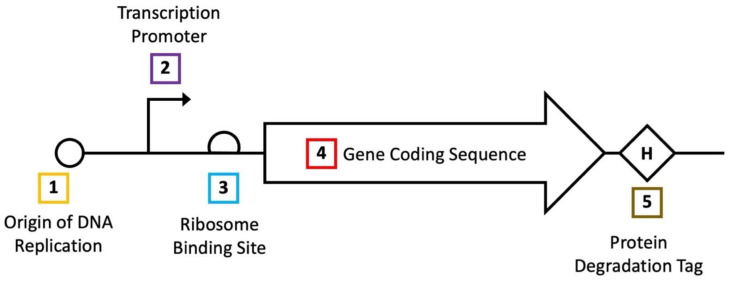
Genetic elements controlling bacterial gene expression. Each of the five elements shown above are commonly used to control a given gene’s steady-state concentration. Altering multiple parameters can change a gene’s expression level by several orders of magnitude.

In living cells, the activity of a gene product is greatly influenced by the steady-state concentration it reaches following expression. In *E. coli*, multiple mechanisms for controlling gene expression are well established, allowing the production of a gene of interest to be tailored to suit an individual project’s needs (Figure 4). For biosensors, functional properties can often be improved by optimizing the expression level of the biosensor itself and the reporter gene it regulates. In contrast to methods involving the direct engineering of biosensor genes, researchers need not develop a customized approach to alter the expression level of a given gene. Improvements resulting from optimizing gene expression alone can, however, be more limited, as this strategy can lead to improvements in the dynamic and operational range but cannot change a sensor’s specificity profile.

In *E. coli*, optimization of gene expression typically considers the rates of four separate processes: DNA replication, RNA transcription, protein translation, and protein degradation. While *E. coli* cells, on average, contain only one copy of genomic DNA, plasmid replication rates can span a much wider range [121]. Thus, by changing the origin of replication regulating a biosensor gene, researchers can control its DNA copy number; characterized origins range from relatively low copy (such as ~5 copies in the case of pSC101) [122] to very high copy (~500–700 in the case of pUC) [123] per cell. Moving down the molecular hierarchy, rates of RNA transcription can also vary widely in *E. coli*. As the transcriptional strength of different promoters has been well studied in this organism, prior investigations enable researchers to select from defined suites of promoters to test different relative rates of transcription [124,125]. Following the production of RNA, one can also control the rate of protein translation, with the most common approach entailing alterations to a gene’s ribosome-binding site (RBS). Analogous to promoters, studies have also generated suites of RBS sequences with measured relative rates of translation initiation [126]. However, in addition to the RBS, a gene’s individual sequence can also affect the rate of translation initiation. Thus, translation optimization can often benefit from empirically testing different RBS variants and predicting their strengths [127]. Additionally, though a less common method, the usage of different codons within a gene’s coding sequence can also be used to alter rates of protein translational elongation [128]. Lastly, a protein’s degradation rate can also be modulated after it is translated through the addition of a degradation tag, which reduces a protein’s steady-state concentration. While several degradation tags have been previously characterized in *E. coli* [129,130,131], this strategy may not prove viable for every project as the addition of a tag may interfere with a given protein’s function. By editing more than one of the processes discussed above, a given gene’s expression level can be tuned across several orders of magnitude.

To date, these approaches have been employed to improve the elements of numerous biosensors, with the facile nature of expression tuning often allowing multiple sensors to be optimized within the context of single studies. For instance, in one 2015 report, Rogers et al. detailed the development of several biosensor systems in *E. coli*, deriving significant improvements from the optimization of plasmid origins [132]. A later study in 2018 similarly worked to improve several biosensor systems in *E. coli* using multiple methods, including the tuning of the promoter and RBS strength for each sensor [133]. Another investigation by Chen et al. in 2018 more deeply explored the effects of promoter engineering, also improving the efficacy of several *E. coli* biosensors [124]. While modulating protein degradation is less common than the aforementioned strategies, multiple studies have appended degradation tags to reporter proteins to improve biosensor functionality [134,135,136,137]. Apart from using previously characterized regulatory elements, gene expression can also be optimized using directed evolution [9,127]. Tuning gene expression is an effective route for shifting biosensor dose–response curves, with most studies choosing either to maximize the induced response or minimize leaky expression in the absence of ligand. However, improvements of one attribute usually come at the expense of the other (e.g., reducing leaky expression also reduces the induced signal). While this drawback limits the benefits that can be derived solely from tuning gene expression, it nonetheless remains a popular strategy for biosensor optimization, particularly in combination with other techniques.

### 3.3. Selection of Reporter System

A major benefit of using biosensors inside living cells for detection is that a single sensor can often be linked to many distinct types of output signals, extending their utility to a wide variety of applications. The type of output is typically dictated by the reporter gene used, with many reporters having been well established in *E. coli*. Fluorescent proteins remain a popular choice, enabling the detection of a fluorescent signal using common laboratory spectroscopic equipment. While green fluorescent protein (GFP) was the first such reporter to be described [138], researchers have since engineered proteins that fluoresce at different wavelengths across the color spectrum, including blue, cyan, green, green–yellow, yellow, orange, red, and far-red fluorescent proteins [139]. A smaller number of fluorescent RNA aptamers have also been reported, capable of producing green- or orange-colored signal. Colored pigments can also be produced using bacterial reporter proteins, with a wide variety of accessible colors available, including red, orange, yellow, green, blue, navy, and purple [140,141]. Luminescent light emission provides another common reporter mechanism, with the activity of bacterial luciferase linked to the expression of the *lux* operon [142]. Apart from spectroscopic reporters, biosensors can also be linked to cell growth through selection markers. In bacteria, antibiotic resistance genes are commonly selected for this purpose [143]; however, the use of auxotrophic markers has also been described [144]. Outside of established systems, recent studies have continued to develop new types of reporters, including genes producing electrical signal [145], gas production [146], and targeted genome editing [147,148].

When deciding on a reporter gene, it is useful to consider the different aspects of the intended application. Firstly, the choice of a reporter can affect the operational range of a biosensor, with enzymatic reporters (such as luciferase or *lacZ*) allowing for more sensitive detection of lower analyte concentrations compared to fluorescent reporters [149,150]. However, fluorescent reporters can sometimes exhibit a broader operational range, and their use may be preferred in instances where a response to higher analyte concentrations is desired [151]. Reporters that produce colored pigments may in turn be preferred for applications spanning longer periods of time, as colored small molecules degrade less rapidly and can accrue more readily compared to detectable proteins. For applications spanning more unique environments outside of the laboratory, additional details of a reporter may also need to be considered. For instance, applications inside living systems often require tissue penetrance of a reporter’s signal. For these applications, luminescent reporters are typically preferred [152], though near-infrared (NIR) fluorescent proteins can also be used [153]. In another example, certain applications (such as soil sample contaminant detection) can require a biosensor to function under anaerobic conditions [154]. As oxygen is required for luciferase activity and for correct maturation of most fluorescent proteins [155], reporters known to function normally within anaerobic environments (such as *lacZ*) are preferred for these applications [154]. In studies using biosensors for metabolic or genetic engineering, the use of selection markers as reporters is typically preferred, although fluorescent proteins can also provide an effective choice particularly when paired with fluorescence-activated cell sorting (FACS) methods [8,156]. When planning a project entailing a novel application of a biosensor, ultimately the options for a reporter can include any gene that can be expressed in bacteria while retaining measurable activity.

In addition to selecting a mechanistic reporter type, it is also useful to consider prior engineering efforts towards improving specific reporter genes. For many of the common reporters detailed above, prior studies have engineered mutant variants exhibiting improved properties. GFP in particular has been subjected to extensive efforts, with improved variants including proteins engineered for enhanced fluorescence [157], more robust protein folding [158], increased thermostability [159], and reduced photobleaching [160]. Improved bacterial luciferase variants have also been developed, with two successive studies yielding variants with significantly brighter luminescent signal [161,162]. In addition to protein engineering, biosensors can also be improved by developing synthetic analogs of their natural substrates. A well-known example of this strategy is the development of X-gal, an artificial substrate for *lacZ* that changes color after enzyme cleavage [163]. Though less commonly used compared to X-gal, alternative *lacZ* substrates have also been developed that produce fluorescent signal following enzyme cleavage [164]. Artificial substrates for firefly luciferase have also been developed to enable increased tissue penetrance of luminescent signal [165], although this strategy has yet to be applied to the bacterial variant of luciferase. Overall, the abundance of prior work to develop and improve genetic reporter systems has made reporter selection an effective route for optimizing many biosensor systems. However, a limitation of this approach is that it cannot be extended to systems where the biosensor and reporter are part of the same protein (such as FRET-based biosensors).

**Figure 5:**
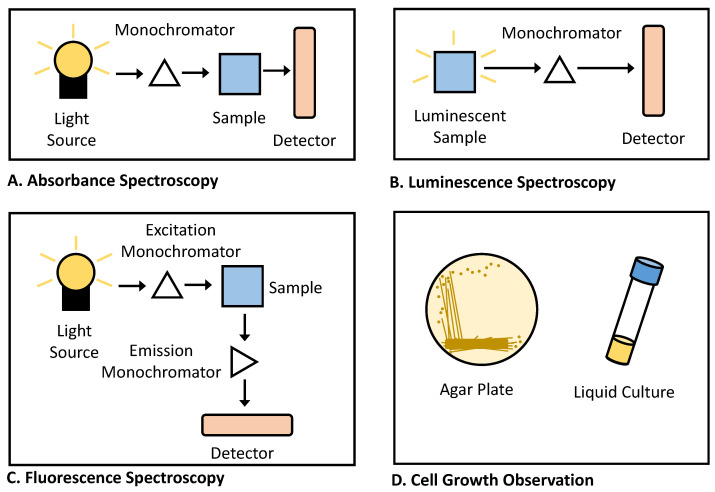
Diagrams of common instrumentation used in bacterial biosensor detection. (**A**) Absorbance spectrophotometers are often used to detect changes of colored dyes, such as the appearance of blue color following cleavage of X-gal by the reporter *lacZ*. (**B**) Luminescence detectors are used to monitor the activity of different types of luciferase reporter genes. (**C**) Fluorimeters are used to monitor the presence of fluorescent proteins (such as GFP). (**D**) Cell growth can be assessed either by counting colonies on solid agar plates or by measuring the absorbance at 600 nm of liquid cultures.

For cellular biosensors, the choice of reporter gene also dictates the instrumentation required for detection. Among the bacterial reporters discussed above, the most commonly used systems each require relatively simple equipment to detect their output signal (Figure 5). For instance, a common absorbance spectrophotometer can be used to quantify colorimetric reporters, such as lacZ or colored pigments (Figure 5A), and a similar device without an added light source can also be used to detect luminescent reporters (Figure 5B). A fluorimeter can in turn be used to quantify signal from fluorescent proteins (Figure 5C). Notably, all three of these spectroscopic functions can be performed across many samples in multiplex using a laboratory plate reader, the use of which is increasingly prevalent in biological labs. For reporters linked to cell growth, bacterial growth can be quantified either by counting colonies on solid agar or by measuring the absorbance of liquid cultures at 600 nm (Figure 5D). When using less common reporter genes, however, more complex or specialized equipment can be required. For example, reporters producing electroactive signal can require a bioelectronic sensing system [145]. In previous work using reporters that function through gas production, a gas chromatograph-mass spectrometer (GC-MS) was required [146]. Consequently, the availability of laboratory equipment is also an important consideration when selecting a biosensor reporter gene.

### 3.4. Incorporation of Additional Genetic Modules

**Figure 6:**
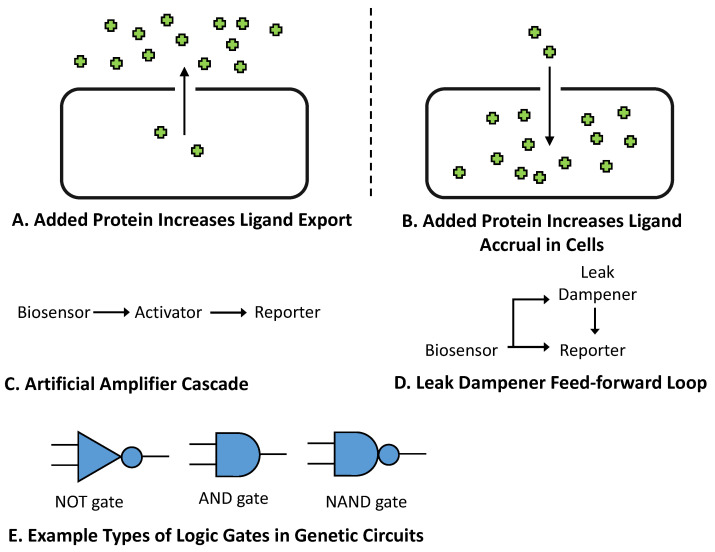
Additional genetic modules for improving biosensor function. (**A**) Genes can be introduced to modify the concentration of ligand compounds inside cells, mediating either increased efflux or (**B**) increased intracellular accrual of analytes. These approaches result in changes to a biosensor’s operational range. (**C**) Activator genes can also be placed between the biosensor and its reporter to construct a gene cascade. The addition of an activator results in an increased biosensor signal and an extended dynamic range. (**D**) Leak dampener genes can also be introduced to regulate reporter genes using a type-1 coherent feed-forward loop. This strategy leads to reduced leaky reporter signal in the absence of analyte ligands, and also extends a biosensor’s dynamic range. (**E**) Additional genes can also be used to change the logic associated with a biosensor’s response. This approach can not only be applied to individual biosensors but can also be applied to link more than one sensor together through a multi-input logic gate.

In addition to biosensors and their associated reporters, additional genes can also be introduced into bacteria to improve sensor function. These added elements can typically be divided in two categories: genes that alter analyte concentrations within the cell (changing biosensor input), and genetic circuits that alter the relationship between the biosensor and its reporter (changing biosensor output). Regarding the first category, changing the steady-state concentration of an analyte inside cells provides a method for shifting a biosensor’s operational range (Figure 6A). In one example, Raman et al. reduced the sensitivity of the tetracycline biosensor *tetR* through the addition of the transmembrane pump *tetA* [137]. In a later work, Johnston et al. reduced the sensitivity of a Rho-based bicyclomycin biosensor in a similar fashion, utilizing the efflux pump *bcmT* [166]. To shift the operational range in the other direction (Figure 6B), Tang et al. incorporated the transporter *fucP* to improve cellular uptake of *d*-arabinose, thereby increasing the sensitivity of the biosensor *araC* [93]. Miller et al. later used a different strategy to increase the sensitivity of the macrolide biosensor *mphR* by adding the phosphotransferase *mphA* into cells, with phosphorylation of macrolide analytes increasing their concentration inside the cells by preventing their outward diffusion [167]. As these examples illustrate, modifying the analyte concentrations inside cells can be used to significantly alter a biosensor’s operational range. However, prior applications of this strategy have each leveraged prior knowledge of characterized proteins that recognize specific analytes. This requirement may consequently limit the use of this approach across other biosensor systems.

Engineered gene cascades provide a versatile method for increasing a biosensor’s output and extending its dynamic range. To construct a cascade, a biosensor is first linked to an activator gene, which is in turn linked to the reporter (Figure 6C). For an activator to be viable for use in a cascade, the production of one activator gene copy must lead to the production or activation of more than one copy of downstream-regulated genes [168]. T7 RNA polymerase provides one such viable activator, with this gene having been incorporated into biosensor-linked cascades both in vitro [169] and in living *E. coli* cells [85,170]. In a recent study, Wan et al. demonstrated that three additional activators (RinA, ECF11, and HrpRS) are effective within bacterial genetic cascades [171]. In this work, the authors linked together all three activators in a single cascade to improve the signal produced by biosensors for lead and mercury [171]. For RNA sensors, ribozymes are similarly used as amplifiers during the construction of aptazymes. While riboswitches directly link a small-molecule ligand to the expression of a reporter, aptazymes instead link ligand binding to the activity of a ribozyme, which is in turn linked to the reporter [172,173]. Genetic cascades provide a highly modular approach for significantly extending a biosensor’s dynamic range, and for shifting the operational range towards increased sensitivity. However, the incorporation of activator cascades often requires additional tuning of expression levels to ensure that the input-output levels of each module fall within their linear regime, thereby avoiding early saturation. Another drawback of this approach is that amplifiers also amplify leaky expression in the absence of analyte signal, though this can be partially offset by decreasing reporter gene expression.

Leaky expression is indeed a significant issue for many biosensor systems, and can often limit many desired applications. The incorporation of a leak dampener circuit provides a modular strategy to help remedy this issue. Leak dampener components are typically introduced as part of a type 1 coherent feed-forward loop, wherein the leak dampener regulates a reporter gene, and a biosensor regulates both the reporter gene and the leak dampener (Figure 6D). To function as a leak dampener, a gene must be able to regulate a reporter through an independent mechanism compared to the biosensor, with lower levels of leak dampener expression resulting in reduced reporter activity [174]. In one example, Ho et al. used the leucine amber suppressor tRNA *supP* as a leak dampener to improve lactate detection using the biosensor *lldR* [174]. This work introduced conditionally silent leucine-to-amber mutations into reporter genes to make their correct translation dependent upon *supP* expression, and notably used a toxic mutagenic reporter system to demonstrate that this approach can achieve undetectable levels of leaky expression in the absence of an inducer [174]. In another study, Greco et al. used a toehold switch (THS) translational regulator as well as a small transcription activating RNA (STAR) as leak dampeners to improve IPTG detection using *lacI* [175]. Expression of the THS and STAR elements was linked to correct translation initiation and transcription initiation, respectively, of the reporter gene, with the structured RNA elements preventing these processes from occurring in the absence of ligand [175]. Another strategy detailed by Fernandez-Rodriguez et al. uses multiple plant proteases as leak dampeners, improving the output of an AND gate built from the biosensors *tetR* and *lacI* [176]. In this work, a cleavable degradation tag was appended to the reporter gene, and expression of the protease led to increased reporter signal via removal of the degradation tag [176]. Similarly to genetic amplifiers, the incorporation of leak dampeners can also increase a biosensor’s dynamic range, and often require additional expression level tuning to ensure that the input-output levels of each module lie within their linear range. In contrast to amplifiers, however, the use of leak dampeners tends to shift the operational range towards reduced sensitivity [174]. Additionally, the use of leak dampeners can often lead to reduced maximum signal [171,174], although this drawback can also be partially remedied by optimizing reporter gene expression levels.

Lastly, additional genetic modules can be introduced to alter the logic associated with a biosensor’s output (Figure 6E). One such genetic part is termed a signal inverter, typically constructed from a constitutive repressor, which is inserted between a biosensor and a reporter within a genetic circuit [177]. The incorporation of this component inverts the logic associated with a biosensor’s output, acting as a single-input NOT gate. To give an example, while the arabinose biosensor *araC* typically produces signal in the presence of arabinose, the addition of an inverter instead results in the production of signal in the absence of this ligand [178]. For a more complex response to analyte ligands, multiple biosensors can also be connected to multi-input logic gates. One such example of an AND logic gate is detailed in a study by Shis and Bennett [179], wherein a split T7 RNA polymerase system is used to make the production of a reporter protein dependent upon the presence of both ligands for two biosensors (*lacI* and *araC*). The same two biosensors were also used by Wang et al. to develop NAND gated logic [180]. In this work, the *hrpRS* system was first used to connect both sensors to form an AND gate. The *hrpRS* genes were subsequently linked to an inverter (the cI repressor) that was linked to a reporter, resulting in NAND logic [180]. In addition to the examples discussed above, other types of multi-input logic gates have also been previously described in *E. coli* [181]. Single-input logic inverters allow for researchers to control whether biosensors produce a response in the presence versus the absence of a ligand, regardless of the original logic of the sensor. The use of a multi-input logic gate can allow for more precise detection of the phenomena of interest, provided, however, that such phenomena are associated with more than one detectable ligand [182].

## 4. Conclusions

The plethora of available engineering approaches provide researchers with considerable latitude for constructing and improving bacterial biosensors, with multiple techniques often combined within the same project for maximal efficacy. Looking ahead, continued advancements in synthetic biology are expected to expand available techniques for biosensor engineering. Advances in continuous directed evolution methods provide one such recent development, with researchers able to rapidly perform numerous cycles of directed evolution by linking them to natural life cycles [183]. Future methods for structure-guided engineering also hold considerable promise, as algorithms for protein structure prediction continue to rapidly improve [184]. Several of the genetic circuit topologies and components described in this review also represent very recent advances [171,174,175], with these approaches being of particular note due to their high modularity and applicability to many biosensor systems.

Improvements in our capacity to optimize bacterial biosensors continue to bring these proteins closer to realizing their potential across many diverse applications. The use of biosensors to improve the biosynthetic yields of biosynthetic pathways is one notable horizon. While yields from bacterial production of many high-value compounds remain below industrially viable levels, biosensors have been used to improve yields by linking pathways to selectable markers for directed evolution [137] and to enable metabolic feedback process control [7]. Bacterial biosensors continue to show promise for the detection of environmental contaminants, and their high specificity and relative ease of use allows for applications in the field [22,185]. The use of biosensors in bacterial cancer therapies demonstrates another exciting application, with multiple sensors shown to enable bacteria to detect and respond to tumors [12]. As effective use of biosensors requires multiple functional properties to be finely tuned, effective methods for biosensor engineering remain essential for realizing the promise of this versatile sensory approach.
